# Critically appraised topic for the most effective and safe treatment for canine generalised demodicosis

**DOI:** 10.1186/s12917-018-1767-7

**Published:** 2019-01-07

**Authors:** Roberta Perego, Eva Spada, Caterina Foppa, Daniela Proverbio

**Affiliations:** Department of Veterinary Medicine (DIMEVET), University of Milan, Via G. Celoria 10, 20133 Milan, Italy

**Keywords:** Canine generalised demodicosis, Demodectic mange, Treatment, Efficacy, Therapeutic protocol, Topical therapy, Systemic therapy

## Abstract

**Background:**

Canine generalised demodicosis is an inflammatory parasitic skin disease caused by an excessive proliferation of *Demodex spp*. Generalized demodicosis is a severe skin disease, that can be life threatening if not treated properly. Many of the current treatment options are not licensed for the treatment of generalised demodicosis, it have a low safety margin and may be poorly efficacious and time-consuming for the owner; there is a need for a safe, efficacious treatment for canine demodicosis. Our objective was to systematically review the literature to determine the most effective and safe topical or systemic therapy for canine generalised demodicosis. Single case reports and case series with fewer than five patients were not reviewed as they were considered to be poor quality evidence. A detailed literature search identified 21 relevant clinical trials and these were critically assessed.

**Results:**

The analysis of the best available evidence on March 5, 2018, suggests that six are the most effective and safe treatments for generalised canine demodicosis including (in alphabetical order): doramectin (oral or parenteral); fluralaner (oral); imidacloprid/moxidectin (topical); ivermectin (oral, not as first choice treatment); milbemycin oxime (oral); and sarolaner (oral). There was insufficient evidence to allow comment on the appropriateness of other treatment protocols for canine generalised demodicosis in this CAT.

**Conclusions:**

In our critical appraisal of the best scientific literature, there is evidence for recommending the use of 6 therapeutic options against demodectic mange. Further, in vivo, controlled, randomized and blinded clinical trials are required, to evaluate new therapies.

## Background

Canine generalized demodicosis in an inflammatory parasitic skin disease, caused by an excessive proliferation of *Demodex spp* mites, which are normal skin commensals in most dogs. According to the extent of the lesions, demodicosis is classified as either localized or generalized. The generalized form can be one of the most severe canine skin disease, and can be life threatening if not treated adequately and promptly [1].

Current available therapeutic options for the treatment of generalised canine demodicosis include many drugs of variable efficacy. Some of these drugs are registered for this use whereas others are not, and some have potentially severe adverse effects [2, 3]. The aim of this critically appraised topic is evaluate the best scientific evidence, to identify the most effective and safe topical or systemic therapy for canine generalised demodicosis.

### Clinical scenario

The patient is a one-year old, intact female, stray Border collie cross. She is malnourished (BCS 3/5), and exhibits intense pruritus and mild enlargement of palpable lymph nodes. On dermatologic examination, there are widespread alopecic patches, crusts with serous-haemorrhagic/purulent exudate, mostly confluent on the head and face, legs, hips and abdomen. Her coat has poor quality and shows scaling, whilst the skin on the abdomen and forelegs is erythematous, with numerous comedones. The case history corresponds to a generalised exfoliative dermatitis associated with a multifocal, itchy, erosive and crusty dermatitis.

Skin cytology, hair plucking, and five deep skin scrapings are performed and the material is examined under the microscope: neutrophil granulocytes with phagocytosed cocci and many *Demodex spp* (adults, larval forms and eggs) are visible. The diagnosis is juvenile-onset generalised demodectic mange associated with secondary bacterial infection; the question is which therapeutic protocol will result in healing of this young stray dog with minimal risk.

### Structured question

In a dog affected with generalised demodicosis, which is the most effective, rapid and safe topical or systemic therapy to ensure complete clinical and parasitological remission?

## Methods

The PUBMED, Web of Science (Science Citation Index Expanded) and CAB Abstract databases were searched on March 5, 2018 using the following string: (dog OR dogs OR canine) and (demodectic mange OR demodicosis) and (therapy OR treatment OR therapeutics OR therapeutic protocol OR efficacy), with no limitations of date and language. We excluded congress proceedings and book chapters.

## Results

Our literature search identified 124, 185 and 485 citations in PUBMED, Web of Science and CAB Abstract databases, respectively. Citations were initially assessed to identify articles reporting original information; review papers were not considered further. Abstracts were then read and pertinent articles were read in full. The bibliography of these articles was examined further for additional pertinent citations. We only selected online available clinical trials testing an effective and safe topical or systemic therapy against generalized demodicosis, with or without control groups, that provided a population of at least 5 naturally affected dogs and where the diagnosis was made performing multiple (three/five) deep skin scrapings of which at least one positive. In the included studies, the number and viability of the *Demodex* spp. microscopically observed at the time of diagnosis and the presence/absence at each subsequent follow up had to be reported to evaluate the efficacy of the therapeutic protocol, that is multiple negative skin scrapings.

After the analysis of all the bibliography, we found 34 pertinent citations. There were 13 duplicated articles and therefore only 21 fulfilled our criteria and were included for further analysis. [4–24] All 21 studies are primary studies, reported between 1983 and 2018, all written in English except one Brazilian study written in Portuguese (Table 1).

**Table 1:** Details of included articles

Reference	Year	Authors	Title
[4]	1983	S.D. Folz et al	Chemotherapeutic treatment of naturally acquired generalized demodicosis.
[5]	1995	W.H. Miller et al	Clinical efficacy of increased dosages of milbemycin oxime for treatment of generalized demodicosis in adult dogs.
[6]	1995	Z. Ristic et al	Ivermectin for treatment of generalized demodicosis in dogs.
[7]	2003	B.R. Holm	Efficacy of milbemycin oxime in the treatment of canine generalized demodicosis: a retrospective study of 99 dogs (1995–2000)
[8]	2005	J. Heine et al	Evaluation of the efficacy and safety of imidacloprid 10% plus moxidectin 2.5% spot-on in the treatment of generalized demodicosis in dogs: results of a European field study.
[9]	2006	E.H. Delayte et al	Eficàcia das lactonas macrocìclicas sistemicas (ivermectina e moxidectina) na terapia da demodicidose canina generalizada.
[10]	2007	L.J. Fourie et al	Efficacy of a novel formulation of metaflumizone plus amitraz for the treatment of demodectic mange in dogs.
[11]	2009	L.J. Fourie et al	Comparative efficacy and safety of two treatment regimens with a topically applied combination of imidacloprid and moxidectin (Advocate) against generalised demodicosis in dogs.
[12]	2010	Murayama et al	Efficacy of weekly oral doramectin treatment in canine demodicosis.
[13]	2000	R. Wagner et al	Field efficacy of moxidectin in dogs and rabbits naturally infested with *Sarcoptes* spp., *Demodex* spp. and *Psoroptes* spp. mites.
[14]	2013	L.J. Fourie et al	Efficacy of a topical application of Certifect (fipronil 6.26% w/v, amitraz 7.48% *w*/*v*, (S)-methoprene 5,63% w/v) for the treatment of canine generalized demodicosis.
[15]	2015	J.H.C. Hutt	Treatment of canine generalized demodicosis using weekly injections of doramectin: 232 cases in the USA (2002–2012).
[16]	2009	T.E. Paterson et al	Treatment of canine-generalized demodicosis: a blind, randomized clinical trial comparing the efficacy of Advocate (Bayer Animal Health) with ivermectin.
[17]	2014	T.E. Paterson et al	Canine generalized demodicosis treated with varying doses of a 2.5% moxidectin + 10% imidacloprid spot-on and oral ivermectin: parasiticidal effects and long-term treatment outcomes.
[18]	2015	L.J. Fourie et al	Efficacy of orally administered fluralaner (Bravecto) or topically applied imidacloprid/moxidectin (Advocate) against generalized demodicosis in dogs.
19	2016	R.H. Six et al	Efficacy of sarolaner, a novel oral isoxazoline, against two common mite infestations in dogs: *Demodex* spp. and *Otodectes cynotis.*
[20]	2016	F. Beugnet et al	Efficacy of oral afoxolaner for the treatment of canine generalised demodicosis
[21]	2017	D.E Snyder et al	Efficacy of lotilaner (Credelio™), a novel oral isoxazoline against naturally occurring mange mite infestations in dogs caused by Demodex spp.
[22]	2018	A.M.Cordero et al	Doramectin in the treatment of generalized demodicosis.
[23]	2018	C. Becskei et al	Efficacy and safety of sarolaner against generalized demodicosis in dogs in European countries: a non-inferiority study.
[24]	2018	L.Duangkaew et al	A field trial in Thailand of the efficacy of oral fluralaner for the treatment of dogs with generalized demodicosis.

The 21 selected articles assess the efficacy of 12 different drugs against canine generalised demodicosis: 4 for topical use (Amitraz [10]; Imidacloprid + Moxidectin [8, 11, 16, 18–20]; Amitraz + Metaflumizone [10, 14]; Amitraz + Fipronil + methoprene [14]) and 8 systemic treatment (Milbemicine Oxime [5, 7, 8]; Doramectin [12, 15, 22]; Moxidectin [9, 13]; Ivermectin [6, 9, 16, 17]; Fluralaner [18, 24]; Afoxolaner [20]; Sarolaner [19, 23]; Lotilaner [21]).

These studies comply with our inclusion criteria and the focussed clinical question, but have very different study designs. We therefore analysed the scientific quality of each study using following parameters to determine the risk of biased evaluation of treatment efficacy, as summarised in Table 2.

- *Levels of evidence:* randomized clinical trials (RCTs) with low risk of bias level IA, medium risk of bias IB and high risk of bias IC. Controlled trials without randomization or blinding with low risk of bias level IIA, medium risk of bias IIB, high risk IIC. Open uncontrolled trials level IV.
- *Randomization:* presence of a method of generation of the randomization sequence and concealment of the allocation of participants to the intervention groups by the people recruiting the participants. Score: 0 (no) – 1 (yes).
- *Blinding:* trial participants were kept unaware of the treatment allocation. Score: 0 (no) – 1 (yes).
- *Similarity between groups:* populations allocated to different groups in the trial share the same characteristics from the beginning and during the study. Score: 0 (no), 1 (deduced form the text), 2 (yes).
- *Equal treatment of groups:* populations allocated to different groups in the trial have been treated similarly except for the therapy. Score: 0 (no), 1 (deduced form the text), 2 (yes).
- *Presence of at least 12 months follow up:* score: 0 (no) – 1 (yes).
- *Group size:* score: 1 (10–20 dogs), 2 (> 20–40 dogs), 3 (> 40 dogs).

**Table 2:** Evaluation of evidence. The studies are categorized as conclusive (total score 8–9), highly suggestive (total score 6–7), suggestive (total score 4–5) and inconclusive (total score ≤ 3)

Reference	Level of evidence	Randomization	Blinding	Group size	Similarity between groups	Equal treatment of groups	Follow up	SCORE	Categorization
[4]	IIB	1	0	3	1	1	0	6	HIGHLY SUGGESTIVE
[5]	IIC	0	0	2	1	1	1	5	SUGGESTIVE
[6]	IIC	0	0	1	1	1	1	4	SUGGESTIVE
[7]	IV	0	0	3	1	1	1	6	HIGHLY SUGGESTIVE
[8]	IB	1	1	3	2	1	0	8	CONCLUSIVE
[9]	IIC	0	0	3	1	1	1	6	HIGHLY SUGGESTIVE
[10]	IIC	0	0	1	1	1	0	3	INCONCLUSIVE
[11]	IIB	1	0	1	1	1	1	5	SUGGESTIVE
[12]	IV	0	0	2	1	1	1	5	SUGGESTIVE
[13]	IIC	0	0	1	1	1	1	4	SUGGESTIVE
[14]	IB	1	1	1	1	1	0	5	SUGGESTIVE
[15]	IV	0	0	3	0	0	0	3	INCONCLUSIVE
[16]	IB	1	1	3	2	1	1	9	CONCLUSIVE
[17]	IB	1	1	2	1	1	1	7	HIGHLY SUGGESTIVE
[18]	IB	1	1	1	1	1	0	5	SUGGESTIVE
[19]	IB	1	1	1	1	1	0	5	SUGGESTIVE
[20]	IB	1	1	1	1	1	0	5	SUGGESTIVE
[21]	IIC	0	0	1	2	1	0	4	SUGGESTIVE
[22]	IC	1	0	1	2	1	1	6	HIGHLY SUGGESTIVE
[23]	IC	1	1	3	1	1	0	7	HIGHLY SUGGESTIVE
[24]	IV	0	0	3	1	1	0	5	SUGGESTIVE

As described in Table 2, at the end of this phase of quality assessment, every study achieved a total score and was thus graded as: conclusive [8, 16], highly suggestive [4, 7, 9, 17, 22, 23], suggestive [5, 6, 11–14, 18–21, 24] or inconclusive [10, 15]. Inconclusive references were immediately excluded.

To emphasise the overall strength of selected studies, we evaluated conclusive, preponderant and suggestive studies for each therapy protocol used, systemic (Table 3) or topical (Table 4), considering the following variables:

- *adverse effects:* None: score 3; Yes, mild and rare (< 10%): score 2; Yes, moderate and common (> 10%): score 1; Yes, severe and common: score 0. The adverse effects only for the references with score 2, 1 and 0 are detailed in Table 5.
- *treatment duration:* More than 4 months: score 1; From 2 to 4 months: score 2; less than 2 months: score 3.
- *efficacy:* Percentage of cured dogs, or percentage of microscopic reduction of mites count. Efficacy < 60%: score 1; > 60 < 80%: score 2; > 80 < 100%: score 3.

**Table 3:** Efficacy of systemic therapies against generalised demodicosis in dogs. References 9 and 22 are repeated twice because two different protocols were tested in the same study

Reference	Therapy	Posology	Adverse effects	Treatment duration	Efficacy
[5]	MILBEMYCIN OXIME	1–2 mg/kg per os every 24 h.	not reported	2	3
[6]	IVERMECTIN	0.6 mg/kg per os every 24 h.	2	2	3
[7]	MILBEMYCINE OXIME	0.5–1.6 mg/kg per os every 24 h.	3	2	3
[8]	MILBEMYCIN OXIME	0.5–1 mg/kg or 1–2 mg/kg every 24 h. for 4 weeks.	1	2	3
[9]	IVERMECTIN tablets	0.6 mg/kg per os every 24 h.	1	1	3
[9]	MOXIDECTIN	0.5 mg/kg per os every 72 h.	1	1	3
[12]	DORAMECTIN	0.6 mg/kg per os weekly.	2	2	2
[13]	MOXIDECTIN	0.4 mg/kg per os every 24 h.	1	2	3
[16]	IVERMECTIN	0.5 mg/kg per os every 24 h.	3	2	3
[17]	IVERMECTIN	0.5 mg/kg per os every 24 h.	1	1	3
[18]	FLURALANER	25 mg/kg per os once.	3	2	3
[19]	SAROLANER	2 mg/kg per os every 30 days for 3 times.	3	2	3
[20]	AFOXOLANER	2.5 mg/kg per os every 2 weeks for 4 times.	3	2	3
[21]	LOTILANER	20 mg/kg per os monthly for 3 times.	3	2	3
[22]	DORAMECTIN	600 μg/kg s.c. once a week	3	2	3
[22]	DORAMECTIN	600 μg/kg per os twice a week	3	2	3
[23]	SAROLANER	2–4 mg/kg per os every 30 days	3	2	3
[24]	FLURALANER	25–50 mg/kg per os once or every 12 weeks	3	2	3

**Table 4:** Efficacy of topical therapies against generalised demodicosis in dogs. Reference 14 is repeated twice because two different protocols were tested in the same study

Reference	Therapy	Posology	Adverse effects	Treatment duration	Efficacy
[4]	AMITRAZ	From 3 to 6 applications at 14-days intervals.	3	2	3
[8]	IMIDACLOPRID+MOXIDECTIN	From 2 to 4 applications at 28-days intervals.	3	2	3
[11]	IMIDACLOPRID+MOXIDECTIN	Group 1 treated at 28-days intervals max 4 times; group 2 at 7-days intervals max 16 times.	2	1	3 (Group 1) 3 (Group 2)
[14]	FIPRONIL + METHOPRENE + AMITRAZ	Group 1 treated at 28-days intervals; group 2 treated at 14-days intervals.	3	2	3 (Group 1) 3 (Group 2)
[14]	METAFLUMIZONE + AMITRAZ	Group 3 treated at 28-days intervals.	3	2	3
[16]	IMIDACLOPRID+MOXIDECTIN	ADV1 treated monthly; ADV2 biweekly; ADV4 weekly.	3	2	1 (ADV1) 2 (ADV2) AND 3 (ADV4)
[17]	IMIDACLOPRID+MOXIDECTIN	ADV1 treated monthly; ADV2 biweekly; ADV4 weekly.	3	1	1 (ADV1) 2 (ADV2) AND 3 (ADV3)
[18]	IMIDACLOPRID+MOXIDECTIN	3 applications at 28-days intervals.	3	2	3
[19]	IMIDACLOPRID+MOXIDECTIN	Weekly from day 0 to day 81.	3	2	3
[20]	IMIDACLOPRID+MOXIDECTIN	4 applications at 14-days intervals.	3	2	3
[23]	IMIDACLOPRID+MOXIDECTIN	From 2 to 6 applications at 7 or 28-days intervals	3	2	3

**Table 5:** Detail of adverse effects of systemic and topical therapies with score 2, 1 and 0. Reference 9 is repeated twice because two different protocols were tested in the same study

Reference	Therapy	Adverse effects score	Type of adverse event	N. of cases
[6]	IVERMECTIN	2	Mild toxicosis	1
[8]	MILBEMICINE OXIME	1	Diarrhoea and neurological signs	7
[9]	IVERMECTIN tablets	1	• Ataxia	3
			• Lethargia	3
			• Sialorrhea	2
			• Disorexia	1
			• Apathy	1
			• Aggressive behavior	1
[9]	MOXIDECTIN	1	• Emesis	8
			• Disorexia	2
			• Anorexia	2
			• Sialorrhea	2
			• Adipsia	1
			• Diarrhea	1
			• Lethargia	4
			• Apatia	2
			• Myoclonia	1
			• Enanthema	1
[11]	IMIDACLOPRID+MOXIDECTIN	2	• Transient erythema	1
			• Scaling	1
[12]	DORAMECTIN	2	Mild ataxia	1
[13]	MOXIDECTIN	1	• Ataxia	2
			• Lethargia, vomiting	1
[17]	IVERMECTIN	1	• Transient neurotoxicosis with bilateral mydriasis, decreased to absent pupillary light response, ataxia and generalized muscle weakness, vomiting, coma (1 dog)	4

## Discussion

There is insufficient evidence for commenting on the use of some evaluated treatment protocols of this CAT. The topical use of amitraz liquid concentrate licensed for the treatment of demodicosis cannot be recommended as there was only a single study reporting this treatment and this had a follow up of less than 12 months [4]. Also both amitraz-based spot-on treatments were evaluated in a single study [14] without follow-up of at least 12 months. With regard to the systemic therapies, the oral administration of moxidectin, evaluated in two unrandomized and unblinded studies [15, 19], cannot be recommended, due to the high percentage (37%) and severity of side effects, whilst the administration of afoxolaner [20] and lotilaner [21], despite appearing very effective easy to administer and safe in the treatment of demodectic mange, cannot be recommended, because there is only one reference reporting use of each molecule and follow up was less than 12 months.

There is evidence for recommending the use of 6 drugs: the first is 10% imidacloprid + 2.5% moxidectin spot-on (licensed for treatment of demodectic mange), that was the most represented topical compound in this CAT; 8 randomized, blinded and controlled (except [11]) clinical trials assessed its efficacy [8, 11, 16–20, 23] with an adequate follow up (except [18–20]); at different application intervals. The efficacy of 10% imidacloprid + 2.5% moxidectin spot-on was demonstrated with monthly application, especially in dogs affected with juvenile generalised demodicosis or with mild forms; its efficacy increased notably with the frequency of application, without reported side effects. The European approved label instructions for the use of 10% imidacloprid + 2.5% moxidectin recommend monthly use with the possibility of increasing the duration and/or the frequency of application especially in severe cases: biweekly and weekly applications do not represent an off-label use of this drug.

Oral administration of milbemycin oxime (licensed for the treatment of demodicosis), was evaluated in 3 clinical trials [5, 7, 8]. This drug was very effective, especially at the highest dosage in severe cases, with moderateor no reported adverse reactions.

The use of doramectin (not licensed for the treatment of demodicosis) was studied in two reports with adequate follow up [12, 22], but only one randomized and blinded trial [22]: subcutaneous injection or oral administration of this macrocyclic lactone are equally effective against demodectic mange in dogs, especially at the dosage of 0.6 mg/kg twice a week. Only 1 subject in one of the two studies [12] showed side effects (transient ataxia).

Only two molecules belonging to the class of isoxazoline, fluralaner and sarolaner (recently registered for the purpose), were adequately studied for the treatment of demodectic mange [18, 19, 23, 24]. Although in three studies [18, 19, 23], adequate follow-up was not performed, there is enough evidence for recommending the use of these novel isoxazolines against canine demodicosis, because of their ease of use, safety (no adverse effects) and efficacy.

Finally, ivermectin has been widely used for the treatment of generalised demodicosis in dogs, although it is not licensed for this purpose and a formulation for dogs is not available. Four clinical trials [6, 9, 16, 17], tested the efficacy of oral administration of injectable ivermectin for cattle [6, 16, 17] and of a pharmaceutical speciality formulated for the purpose [9]; to achieve clinical and parasitological cure treatment duration was more than 3 months, but the efficacy was excellent. However mild and moderate adverse reactions occurred in these reviewed trials (three out of four) and specials attention must be paid in collies dogs. Despite oral ivermectin is extremely effective against Demodex spp. and there is good evidence for its use, because of the common side effects is not recommended as first choice treatment.

## Conclusion

This critically appraised topic, based on evaluation of the current evidence, demonstrates that only 6 treatment regimens can be recommended for use against canine generalised demodicosis (moxidectin + imidacloprid spot-on; oral milbemycin oxime, oral or parenteral doramectin, oral fluralaner oral sarolaner and, not as first choice treatment, oral ivermectin). The remaining therapies, although sometimes effective, have not been adequately evaluated. For future studies, we recommend in vivo, randomized, blinded and controlled clinical trials, providing a post treatment follow up of 12 months, so that relapses are immediately detected. The efficacy of any new compounds should be tested properly, to help veterinarians in the cure of this widespread dermatological disease.
